# Radioactive ^125^I Seed Inhibits the Cell Growth, Migration, and Invasion of Nasopharyngeal Carcinoma by Triggering DNA Damage and Inactivating VEGF-A/ERK Signaling

**DOI:** 10.1371/journal.pone.0074038

**Published:** 2013-09-10

**Authors:** Yunhong Tian, Qiang Xie, Yunming Tian, Ying Liu, Zuoping Huang, Cundong Fan, Bing Hou, Dan Sun, Kaitai Yao, Tianfeng Chen

**Affiliations:** 1 Cancer Research Institute, Southern Medical University, Guangzhou, Guangdong Province, People’s Republic of China; 2 Department of Oncology, Armed Police Hospital of Guangdong Province, Guangzhou, Guangdong Province, People's Republic of China; 3 Department of Pathology, Medical College of Jinan University, Guangzhou, Guangdong Province, People’s Republic of China; 4 State Key Laboratory Oncology in Southern China, Guangzhou, Guangdong Province, People’s Republic of China; 5 Department of Radiation Oncology, Cancer Center of Sun Yat-Sen University, Guangzhou, Guangdong Province, People’s Republic of China; 6 Department of Chemistry, Jinan University, Guangzhou, Guangdong Province, People’s Republic of China; China Medical University, Taiwan

## Abstract

Although radiotherapy technology has progressed rapidly in the past decade, the inefficiency of radiation and cancer cell resistance mean that the 5-year survival rate of patients with nasopharyngeal carcinoma (NPC) is low. Radioactive ^125^I seed implantation has received increasing attention as a clinical treatment for cancers. Vascular endothelial growth factor-A (VEGF-A) is one of the most important members of the VEGF family and plays an important role in cell migration through the extracellular-signal-regulated kinase (ERK) pathway. Here we show that radioactive ^125^I seeds more effectively inhibit NPC cell growth through DNA damage and subsequent induction of apoptosis, compared with X-ray irradiation. Moreover, cell migration was effectively inhibited by ^125^I seed irradiation through VEGF-A/ERK inactivation. VEGF-A pretreatment significantly blocked ^125^I seed irradiation-induced inhibition of cell migration by recovering the levels of phosphorylated ERK (p-ERK) protein. Interestingly, *in vivo* study results confirmed that ^125^I seed irradiation was more effective in inhibiting tumor growth than X-ray irradiation. Taken together, these results suggest that radioactive ^125^I seeds exert novel anticancer activity by triggering DNA damage and inactivating VEGF-A/ERK signaling. Our finding provides evidence for the efficacy of ^125^I seeds for treating NPC patients, especially those with local recurrence.

## Introduction

Radiotherapy technology has rapidly advanced in the past decade; however, it remains inefficient, and cancer cells can become resistant. As a result, the 5-year survival rate of patients with nasopharyngeal carcinoma (NPC) is about 70% [1]. The complications of radiotherapy (e.g. radiation-induced brain injury) severely affect patient quality of life and can be a significant source of morbidity [2]. Local recurrence is still a major cause of mortality and morbidity in the advanced stages of disease and remains a challenging issue in NPC [3]. Therefore, it is important to explore new effective treatment modalities for NPC patients.

^125^I seeds have an average energy of 27.4-31.4 keV, and their valid radius is 1.7 cm in tissue; they are the most selected radioactive source for permanent implantation. With increasing distance from the radioactive source, gamma ray energy decreased rapidly. When the low-energy ^125^I seeds are implanted, the gamma rays are concentrated in the immediate surrounding tissues, sparing adjacent normal structures and medical personnel [4,5]. Because of its high precision and low complication rate, radioactive ^125^I seed implantation has been widely applied in treatment of cancers, such as recurrent colorectal cancer [6,7], head and neck carcinoma and NPC [4,5]. Several studies have demonstrated that ^125^I seed irradiation is more effective in inducing cell apoptosis in PANC-1 pancreatic [8] and CL187 colonic cells [9,10].

However, few articles are available regarding the biological effects of ^125^I seed irradiation on NPC cell lines. Furthermore, there are a limited number of reports about the effects of ^125^I seed irradiation on cancer cell migration and invasion.

Vascular endothelial growth factor A (VEGF-A) is an important VEGF family member that is essential for cell proliferation and migration [11–14]. Overexpression of VEGF-A can augment cell proliferation and migration through extracellular-signal-related kinase (ERK) signaling. VEGF-A overexpression is associated with poor prognosis in cancer patients [15–17]. A previous report described a post-radiation increase in VEGF-A enhanced glioma cell motility *in vitro* [18]. In this study, we evaluated the effects of radioactive ^125^I seeds on NPC cell growth and migration. Our results demonstrate that radioactive ^125^I seeds more effectively inhibit NPC cell growth by inducing apoptosis due to DNA damage compared with X-ray irradiation. Moreover, cell migration was effectively inhibited by ^125^I seed irradiation through inactivation of VEGF-A/ERK signaling. Pretreatment of cells with VEGF-A significantly blocked ^125^I seed irradiation-induced inhibition on cell migration by recovering phosphorylated ERK (p-ERK) protein levels. Interestingly, the *in vivo* study results confirmed that ^125^I seed irradiation was more effective in inhibiting tumor growth than X-ray irradiation. Taken together, these results suggest that radioactive ^125^I seeds exhibit novel anticancer activity by triggering DNA damage and inactivating the VEGF-A/ERK signaling. These findings provide evidence for the efficacy of ^125^I seeds for the treatment of patients with NPC, especially those with local recurrence.

## Materials and Methods

### 2.1 Cell culture and reagents

CNE2 cell lines were available at the Cancer Institute of Southern Medical University (Guangzhou, China) and were originally purchased from the American Type Culture Collection (ATCC). The authenticities of cell lines in our study have verified with DNA fingerprinting. Cells were maintained in RPMI 1640 media supplemented with 10% fetal bovine serum (FBS, Hyclone, Utah, USA) and antibiotics (100 IU/ml penicillin and 100 mg/ml streptomycin) at 37^o^C under a humidified atmosphere of 95% air and 5% CO_2_. VEGF-A was obtained from R&D Systems (Minnesota, USA). To investigate the role of reactive oxygen species (ROS) in ^125^I seed irradiation, 5 mM glutathione (GSH, Sigma-Aldrich, Missouri, USA) was added 2 hours before irradiation.

### 2.2 Treatments of NPC cells with ^125^I seeds and X-ray irradiation

**Figure 1:**
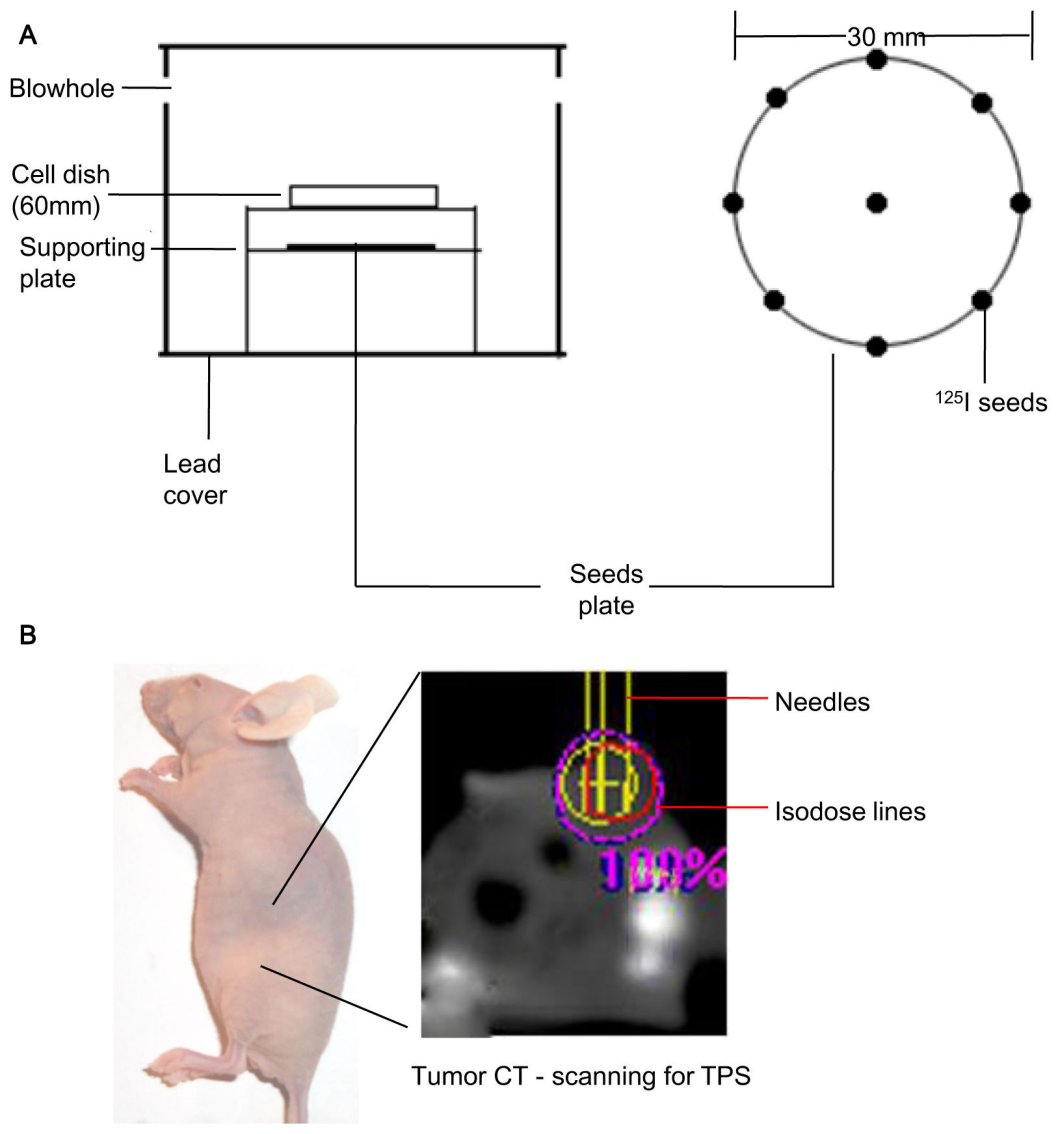
Irradiation models of ^125^I seeds. (A) *In vitro* model, eight ^125^I seeds were evenly taped around a 30-mm diameter circumference, with one ^125^I seed placed in the center. (B) *In vivo* model, a transverse CT scanning was performed on mice, and the dose distribution was calculated by TPS and the GTV (the red circle) should be kept inside the 90% isodose curve (blue one) in every plan. 8 Seeds were implanted into different position by the needle (the three yellow vertical lines) according to TPS.

In-house ^125^I seeds were obtained from Beijing Atom and High Technique Industries Inc. (Beijing, China). *In vitro* irradiation was carried out as depicted in Figure 1A [9]. The absorbed dose was also measured and verified: 44, 92, 144 and 204 hours were required for doses of 2, 4, 6 and 8 Gy, respectively [10]. X-ray irradiation was performed at the Department of Radiotherapy, Armed Police Corps Hospital of Guangdong Province, using an Elekta precise treatment system (Stockholm, Sweden) with a clinically calibrated irradiation field of 10 × 10 cm.

### 2.3 Colony formation and MTT assay

We plated an appropriate number of cells to obtain the correct data for plating efficiency (PE) for nonirradiated controls. PE was calculated as follows: number of colonies / number of seeded cells × 100%. The CNE2 cells exposed to radiation were seeded at 500, 1000, 2000, 4000, or 8000 cells in a 100-mm culture plate, respectively for a total dose of 0, 2, 4, 6, or 8 Gy, respectively. Following irradiation, the cells were incubated for 12 days at 37^o^C in a 5% CO_2_ environment to allow colony formation. Surviving fractions (SFs) were calculated following formula: SF = number of colonies / number of seeded cells × PE. The dose-survival curve was fitted based on the single-hit multi-target theory formula: SF =1 -(1 -e^-D/D0^)^N^; log N = D_q_ / D_0_. Cell viability was determined by measuring the cells’ ability to transform thiazolyl blue tetrazolium bromide (MTT) to a purple formazandye as previously described [19]. Briefly, after irradiation, 20 μl MTT solution (5 mg/ml in phosphate-buffered saline [PBS]) was added to each well in 96-well plate and incubated for 5 hours. The medium was replaced with 200 μl/well of dimethyl sulfoxide (DMSO) to dissolve purple formazan. The color intensity of the formazan solution, which is positively correlated with cell viability, was measured with a microplate spectrophotometer (VSERSA Max, Molecular Devices, California, USA) at 570 nm.

### 2.4 EdU assay

Cell proliferation was measured by 5-ethynyl-2´-deoxyuridine (EdU) assay using an EdU assay kit (Ribobio, Guangzhou, China) according to the manufacturer’s protocol. Briefly, CNE2 cells exposed to radiation were seeded in a 60-mm culture plate. 24 hours later, EdU was added. The cells were then fixed with 4% formaldehyde for 15 minutes and treated with 0.5% Triton X-100 for 20 minutes at room temperature. Finally, the DNA contents of each well were stained with Hoechst 33342 and viewed under a microscope (Nikon, Tokyo, Japan).

### 2.5 Detection of oxidative stress intracellular ROS

For intracellular ROS analysis, CNE2 cells were irradiated at a various doses; 24 hours later, cells were loaded with 10 μM DCF-DA (Sigma-Aldrich, Missouri, USA), incubated at 37^o^C for 30 minutes, and immediately analyzed by flow cytometry (BD Biosciences, California, USA). H_2_O_2_ was used as a positive control.

### 2.6 Annexin V–PI apoptosis and caspase-3 activity assay

Cells exposed to irradiation were harvested 24 hours after irradiation. Annexin V–PI apoptosis assay was performed according to the Alexa Fluor^®^ 488 annexin V/Dead Cell kit protocol (Invitrogen, California, USA). Cells were analyzed by BD FACSCAria™ (BD Biosciences, California, USA). Caspase-3 activity was measured using a Caspase-3 Activity Assay kit (Beyotime Institute of Biotechnology, Jiangsu, China) following the manufacturer’s instructions. Cells incubated 48 hours after irradiation at various doses were lysed with lysis buffer (100 μl per 2 × 10^6^ cells) for 15 minutes on ice following washing with D-Hank’s medium. Then cell extracts were mixed with Ac-DEVD-pNA substrate and incubated at 37°C for 2 hours prior to colorimetric measurement of p-nitroanilide product at 405 nm. The values of treated samples were normalized to untreated controls to determine the fold change in caspase-3 activity.

### 2.7 TUNEL assay

Cells were cultured in chamber slides 24 hours after irradiation and were fixed with 3.7% formaldehyde and permeabilized with 0.1% Triton X-100 in PBS. Then, the cells were incubated with 100 μl/well TUNEL reaction mixture for 1 hour and 1 μg/ml of DAPI for 15 minutes at 37^o^C, respectively. The cells were then washed with PBS and examined under a microscope (Nikon, Tokyo, Japan).

### 2.8 Wound healing assay

At 24 hours after irradiation at a dose of 4 Gy, cells were seeded in a 60-mm culture plate. Similar sized wounds were made by scraping a conventional 10-μl micropipette tip across the monolayer. The distance between the wound edges was measured immediately after wounding and 24 and 48 hours later. The total distance migrated by wounded CNE2 cells was evaluated using Adobe Photoshop and is expressed as a percentage of the initial wound distance.

### 2.9 Transwell and Boyden chamber assay

Transwell and Boyden assays were performed using 24-well transwell permeable supports with or without Matrigel coating (6.5-mm diameter, 10-µm thickness, 8-µm pores; Corning, New York, USA). Briefly, cell suspensions were obtained 24 hours after irradiation at a total dose of 4 Gy. Then, 100 µl containing 10^6^ cells in serum-free RPMI 1640 media were added to the upper chamber and 500 µl RPMI 1640 media with 10% FBS was added to the lower chamber. Cells were incubated for 48 hours at 37°C, and the membrane was stained with crystal violet to calculate the average number of migrated cells [20]. To investigate the effect of VEGF-A on migration, the growth factor was added (20 ng/ml) prior to irradiation, and cells were harvested 24 hours later for transwell assays.

### 2.10 Flow cytometric analysis

Cells were harvested 24 hours after X-ray irradiation and ^125^I seeds treatments. Cells were washed with cold PBS and fixed overnight in cold 70% ethanol. Fixed cells were washed with PBS, resuspended in 100 μl RNase A (250 μg/ml), incubated for 30 minutes at 37°C. Finally, 50 μg/ml PI was added, and the mixtures were incubated at room temperature in the dark for 30 minutes until PI-detection with BD FACSCAria™ (BD Biosciences, California, USA).

### 2.11 Immunofluorescent assay

Cells seeded on slides were washed, fixed and permeabilized for 10 minutes. A primary antibody against-VEGF-A (1:200, Santa Cruz Biotechnology, California, USA) and Alexa Fluor 488-conguated secondary antibody (1:500, Invitrogen, California, USA) were used. The cell nuclei were stained with 4’, 6’-diamino-2-phenylindole (DAPI) (Invitrogen, California, USA). The images were recorded by fluorescence microscopy with a Nikon Eclipse 80i microscope (Nikon, Tokyo, Japan). The primary antibodies were omitted for negative-control staining.

### 2.12 Western blotting analysis

Protein from cells or tumor tissues were mixed with loading buffer and heated at 70°C for 10 minutes. They were then loaded on sodium dodecyl sulfate (SDS)-polyacrylamide gels at 30 µg per lane. After electrophoresis the proteins were transferred to polyvinylidene fluoride (PVDF, Millipore, Massachusett, USA). Membranes were blocked for 2 hours in 5% bovine serum albumin (BSA) and incubated overnight at 4^o^C with the SP rabbit polyclonal antibody (1:1000, Santa Cruz Biotechnology, California, USA). The blots were then incubated with horseradish peroxidase (HRP)-conjugated secondary antibody (1:1,000, Santa Cruz Biotechnology, California, USA). Finally, bands were visualized by enhanced chemiluminescence (ECL, Thermo Scientific Pierce, Illinois, USA).

### 2.13 Enzyme-linked immunosorbent assay (ELISA) for extracellular VEGF-A levels

CNE2 cells were seeded into 6-well plate at a density of 1 × 10^5^ cells/well for 24 hours and then irradiated at various doses. Culture supernatants were collected 24 hours later and determined by ELISA according to the manufacturer’s protocol (Boster, Wuhan, China).

### 2.14 *In vivo experiments*

Female BALB/c nude mice (4-6 weeks old) were purchased from the Model Animal Research Center of Nanjing University. According to the United States Public Health Service (USPHS) Guide for the care and use of laboratory animals and China animal welfare regulations, the *in vivo* experiments were in strict agreement with the institutionally approved protocol. All experiments were approved by the animal care committee of Southern Medical University. Animals were injected subcutaneously (s.c.) with cells into the right hind limb (5 × 10^6^ cells/100 µl). After 2 weeks, mice whose tumor volumes reached approximately 200 mm^3^ were randomly divided into three groups. For treated group, mice were irradiated by X-ray or implanted with ^125^I seeds at a total dose of 20 Gy (2 Gy/day × 10 Fractions for X-ray irradiation). In order to provide an equal total dose, CT-scanning was performed on every nude mouse. Precise calculation of the number of seeds to be implanted was completed using the treatment planning system (TPS) (RT-RSI, Beijing Atom and High Technique Industries Inc., Beijing, China), which was often used to obtain the parameters required for the planning and the choice of treatment parameters such as number of beams, field size, and so on (Figure 1B). We implanted 8 ± 0.5 seeds in the tumor center of anesthetized and sterilized animals. Body weight was measured every 3 days. Animals were euthanized on day 15 after treatment, and tumors were dissected and weighted. Then, immunohistochemistry (IHC) and western blotting for VEGF-A was performed in xenograft tumor samples.

### 2.15 Statistical analysis

Statistical analysis was performed with SPSS statistical package (v 15.0). *In vitro* experiments were repeated three times and data are presented as the mean ± standard deviation (SD). Statistical differences among groups were assessed with one-way analysis of variance (ANOVA), with *p* values of less than 0.05 considered statistically significant.

## Results

### Radioactive ^125^I seeds are more effective than X-ray in inhibiting NPC cell growth, migration, and invasion than X-ray

**Figure 2:**
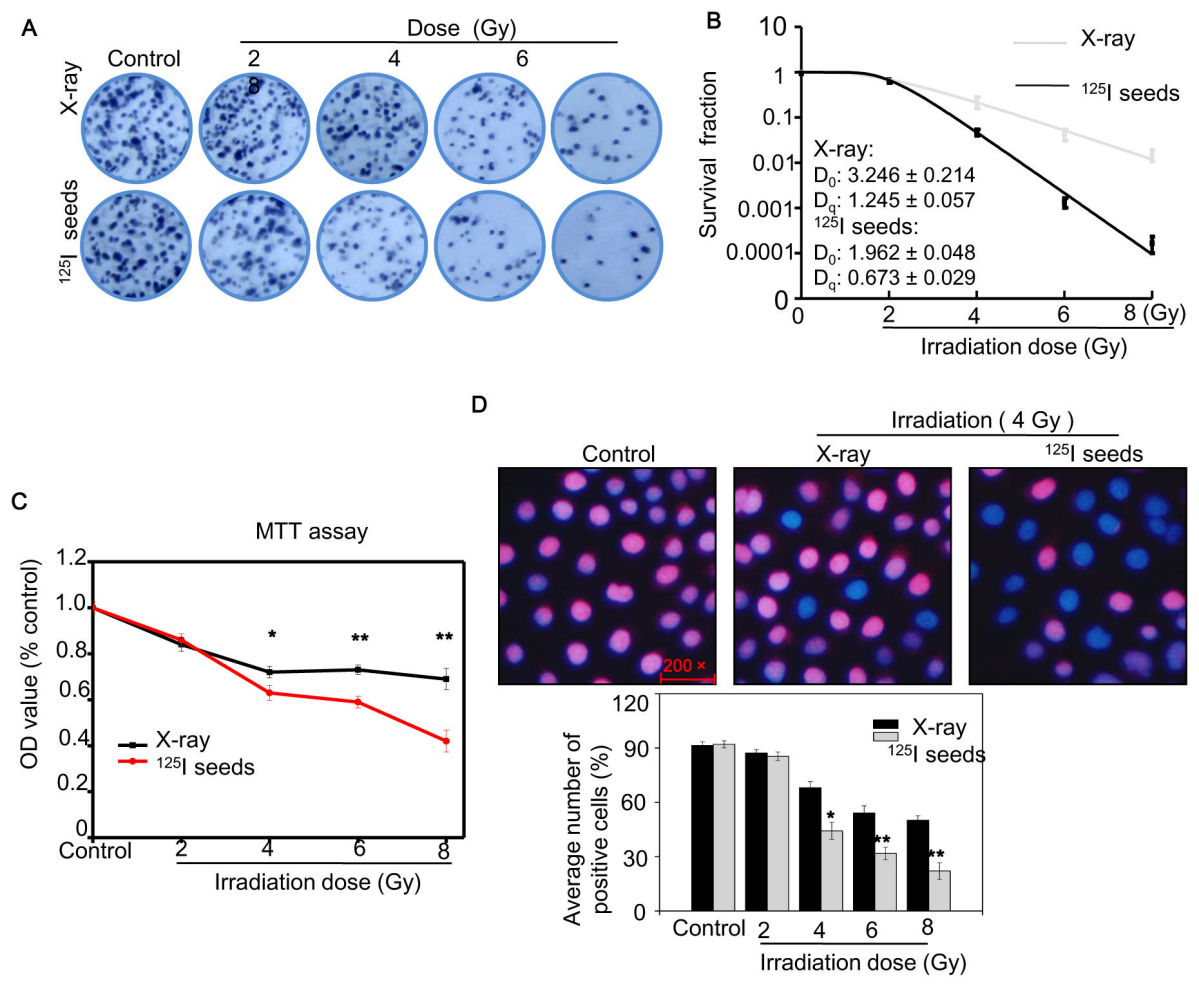
^125^I seed and X-ray irradiation inhibit CNE2 cell proliferation. (A) Representative pictures of colony formation of cells exposed to ^125^I seed and X-ray at various doses for 14 days. (B) The survival fraction of colony formation assay was fitted by single-hit multitarget theory formula. (C) Cell viability of CNE2 cells treated for irradiation was examined by MTT assay. (D) Cell proliferation of CNE2 cells treated for irradiation was examined by EdU assay. Significant difference between ^125^I seed and X-ray groups under the same dose is indicated by * *P*<0.05 and ** *P*<0.01.

Colony formation and MTT assay were employed to examine the effects of ^125^I seeds and X-ray irradiation on CNE2 cell growth. The results showed that colony formation ability was significantly inhibited by ^125^I seeds and X-ray irradiation in a dose-dependent manner (Figure 2A). The relative SF of cells exposed to ^125^I seed irradiation was significantly lower than that of X-ray irradiation (*P* < 0.05). When dose-survival curves were fitted with the single-hit multi-target theory formula, the parameters D_0_ and Dq could be used to characterize radiosensitivity, where the larger values indicate greater radioresistance. As shown in Figure 2B, D_0_ and D_q_ of ^125^I seed irradiation were both significantly lower than those of X-ray irradiation (*P* < 0.05). The results measured by MTT assay also indicated that the viability of cells exposed to various doses of ^125^I seeds was lower than those exposed for the same dose of X-ray (Figure 2C). Moreover, cell proliferation as measured by EdU was significantly inhibited by ^125^I seeds in a dose-dependent manner (Figure 2D). Taken together, these results reveal that cell growth inhibition by irradiation is dependent on the doses, and more importantly, ^125^I seeds irradiation is more effective in inhibiting NPC cell growth than X-ray.

**Figure 3:**
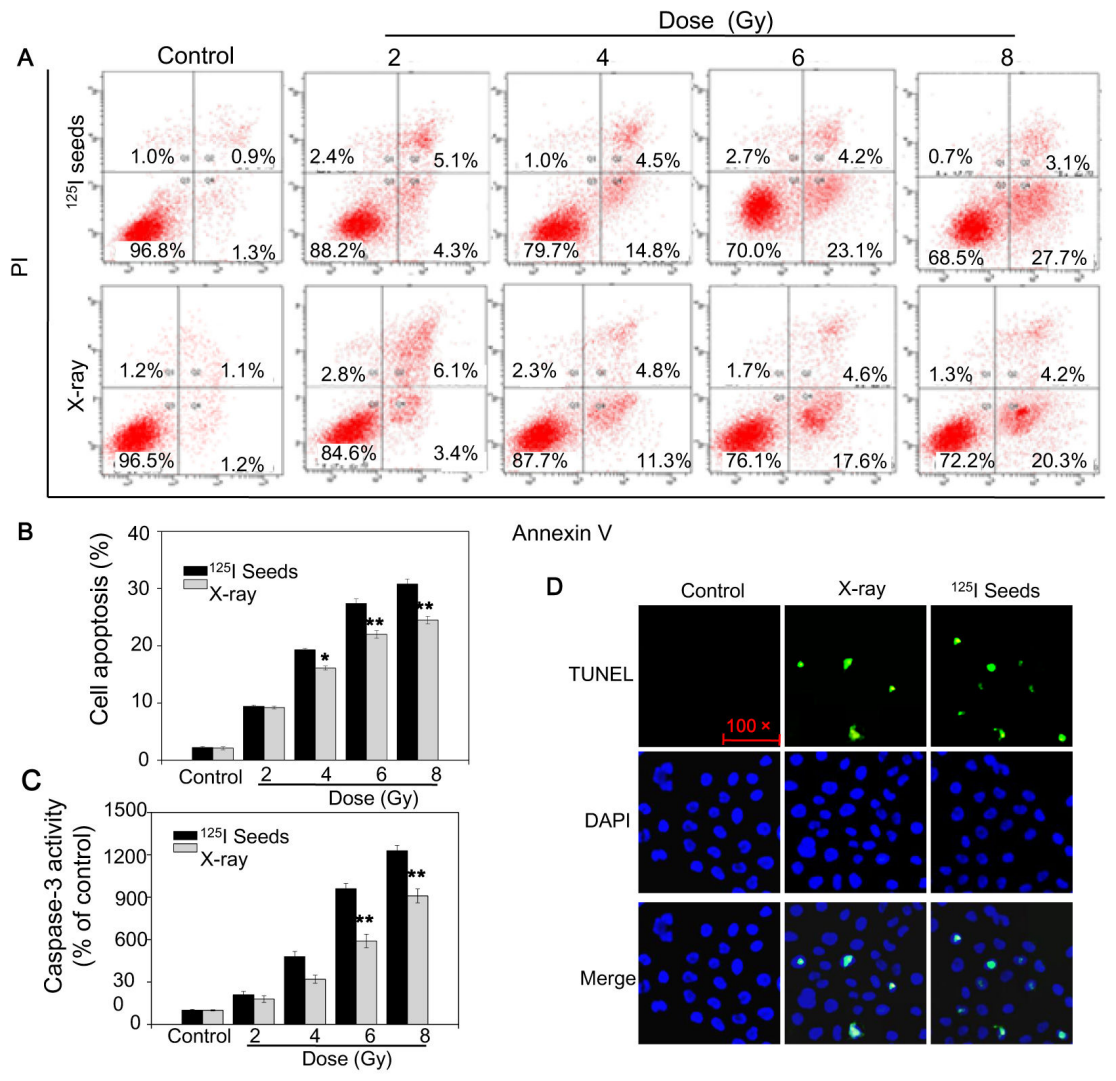
^125^I seed irradiation induces apoptosis of CNE2 cells. Apoptosis was examined by Annexin V–PI co-staining flow cytometric analysis (A, B), caspase-3 activity assay (C) and TUNEL assay (D). Cells exposed to irradiation were harvested 24 hours after irradiation. Then, apoptosis was measured. Significant difference between ^125^I seed and X-ray groups under the same dose is indicated by * *P*<0.05 and ** *P*<0.01.

To examine the modes of cell death after ^125^I seed irradiation, annexin V–PI apoptosis assays were performed. The results showed that apoptotic cell death was markedly induced by X-ray and ^125^I seed irradiation in a dose-dependent manner. However, compared with X-ray irradiation, ^125^I seed irradiation induced a higher percentage of apoptosis (Figure 3A, B). We also investigated whether irradiation-induced apoptosis was related to caspase-3 activation. Interestingly, the results showed that caspase-3 activity increased 24 hours after X-ray and ^125^I seed irradiation in a dose-dependent manner and that ^125^I seed irradiation had a greater effect than X-ray (Figure 3C). Apoptosis was further characterized with TUNEL assays. After exposure to ^125^I seeds, CNE2 cells exhibited enhanced apoptotic features, such as DNA fragmentation and nuclear condensation (Figure 3D). These results suggest that ^125^I seed irradiation is more potent in inducing cancer cell apoptosis.

**Figure 4:**
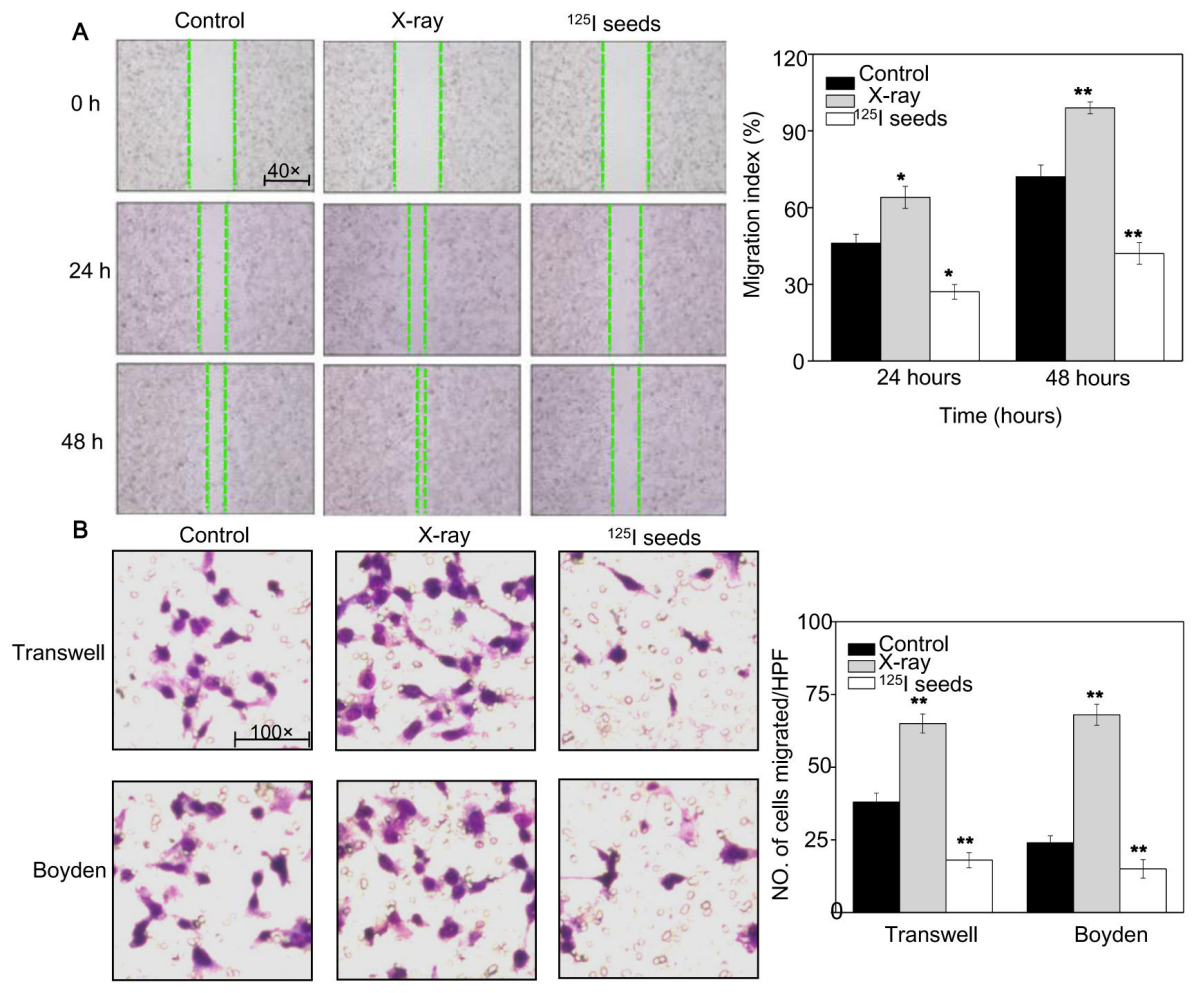
Effects of ^125^I seed irradiation on cells migration and invasion. Cell suspensions were obtained 24 hours after irradiation at a total dose of 4 Gy, and then they were plated in 60-mm culture plate. (A) Wound healing assay was performed 12 hours after plating. The total distance migrated by wounded cells was expressed as percentage of initial distance. (B) The inhibition of cell invasion was measured by transwell and Boyden chamber assay. The number of cells was counted to calculate the average number of migrated cells. Data are presented as mean ± SD (n = 3). *P<0.05, ** *P*<0.01 versus the control group.

We also compared NPC cell migration and invasion between X-ray and ^125^I seed irradiation conditions. As shown in Figure 4A, the migration index of ^125^I irradiation decreased from 47.9% and 70.1% (control) to 30.1% and 42.7% after 24 and 48 hours irradiation, respectively. However, greater NPC cell migration was observed in the X-ray irradiation group at both 24 hours and 48 hours after irradiation. Moreover, transwell and Boyden assays were performed to investigate the effects of both treatments on invasion (Figure 4B). As expected, cell invasive ability decreased significantly after ^125^I seed irradiation, but lower effects were observed in cells exposed to X-ray irradiation. Taken together, the results support the hypothesis that ^125^I seed irradiation more effectively inhibits cancer cell migration and invasion.

### Radioactive ^125^I seeds trigger DNA damage to induce NPC cell apoptosis and G2/M arrest

**Figure 5:**
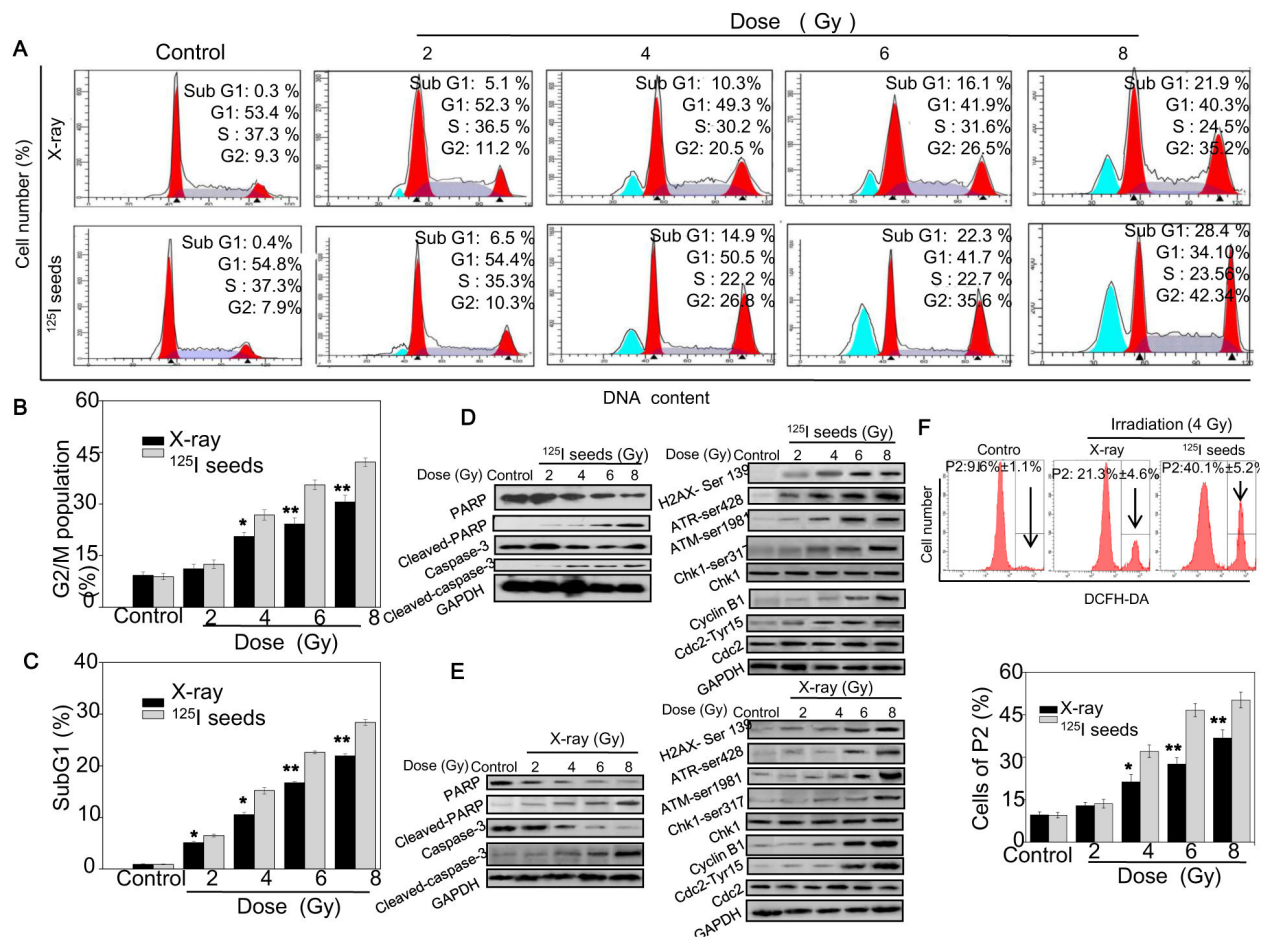
Induction of G2/M arrest and ROS generation by ^125^I seed irradiation. The cells were exposed to ^125^I seed and X-ray irradiation at various doses. 24 hours after irradiation, the effects of ^125^I seed on the cell cycle distribution of CNE2 cells was examined by flow cytometric analysis (A). Quantification of the percentage of G2/M phase (B) and apoptosis reflected by Sub G1(C). (D, E) Effects of ^125^I seed on the expression levels of apoptosis and cell cycle arrest-associated proteins was analyzed by western blotting. (F) The level of ROS was measured by flow cytometry. Data are presented as mean ± SD (n = 3). Significant difference between ^125^I seed and X-ray groups under the same dose is indicated by * *P*<0.05 and ** *P*<0.01.

To clarify the mode of cell death induced by ^125^I seed irradiation, treated cells were examined by flow cytometric analysis. Figure 5A shows the representative DNA distribution histograms of CNE2 cells. They demonstrate dose-dependent increases in G2/M cell populations in cells exposed to X-ray and ^125^I seed irradiation for 24 hours, with no significant changes in S and G0/G1 phase. Moreover, ^125^I seed irradiation induced a higher percentage of G2/M arrest than X-ray (Figure 5B). In addition, exposure of cells to ^125^I seeds resulted in a significantly greater increase in apoptotic cell number than X-ray, as reflected by the increase in sub-G1 peaks. As shown in Figure 5C, the proportion of apoptotic cells exposed to ^125^I seeds increased from 0.9% to 29.8%. At 4 Gy, the proportion of apoptotic cells exposed to ^125^I seeds was 14.9%, compared to 10.3% for X-ray. Western blotting was employed to confirm apoptosis in CNE2 cells at the protein level. As shown in Figure 5D, ^125^I seeds induced poly ADP ribose polymerase (PARP) and caspase-3 cleavage in a dose-dependent manner, indicating that seed irradiation activates caspase-mediated apoptosis.

Previous studies have demonstrated that cells have devolved mechanisms to regulate cell cycle progression and minimize the harmful impact of irradiation, and DNA damage response pathways have evolved to monitor genome integrity [21]. ATM and ATR are the major kinases of the core molecular sensor, and can be recruited in response to DNA damage [22,23], followed by the activation of down-stream signaling molecules, finally resulting in cell cycle arrest or apoptosis. As expected, ^125^I seeds treatments caused an obvious DNA damage in a dose-dependent manner and was accompanied by up-regulation of phosphorylation of ATM (Ser 1981), ATR (Ser 428), Chk1 (Ser 317), Cyclin B1, and Cdc2 (Tyr 15) but did not affect the expression levels of total Chk1 or Cdc2 (Figure 5E). Other studies have shown that ROS play an important role in cancer therapy. Therefore, we measured ROS 24 hours after irradiation. DCF-DA staining revealed that ROS levels were markedly increased 24 hours after ^125^I seed irradiation (Figure 5F). Taken together, these results support the concept that ^125^I seeds directly or indirectly trigger DNA damage to induce NPC cell apoptosis and G2/M arrest.

### Radioactive ^125^I seeds suppress cell migration by inactivating VEGF-A/ERK signaling

**Figure 6:**
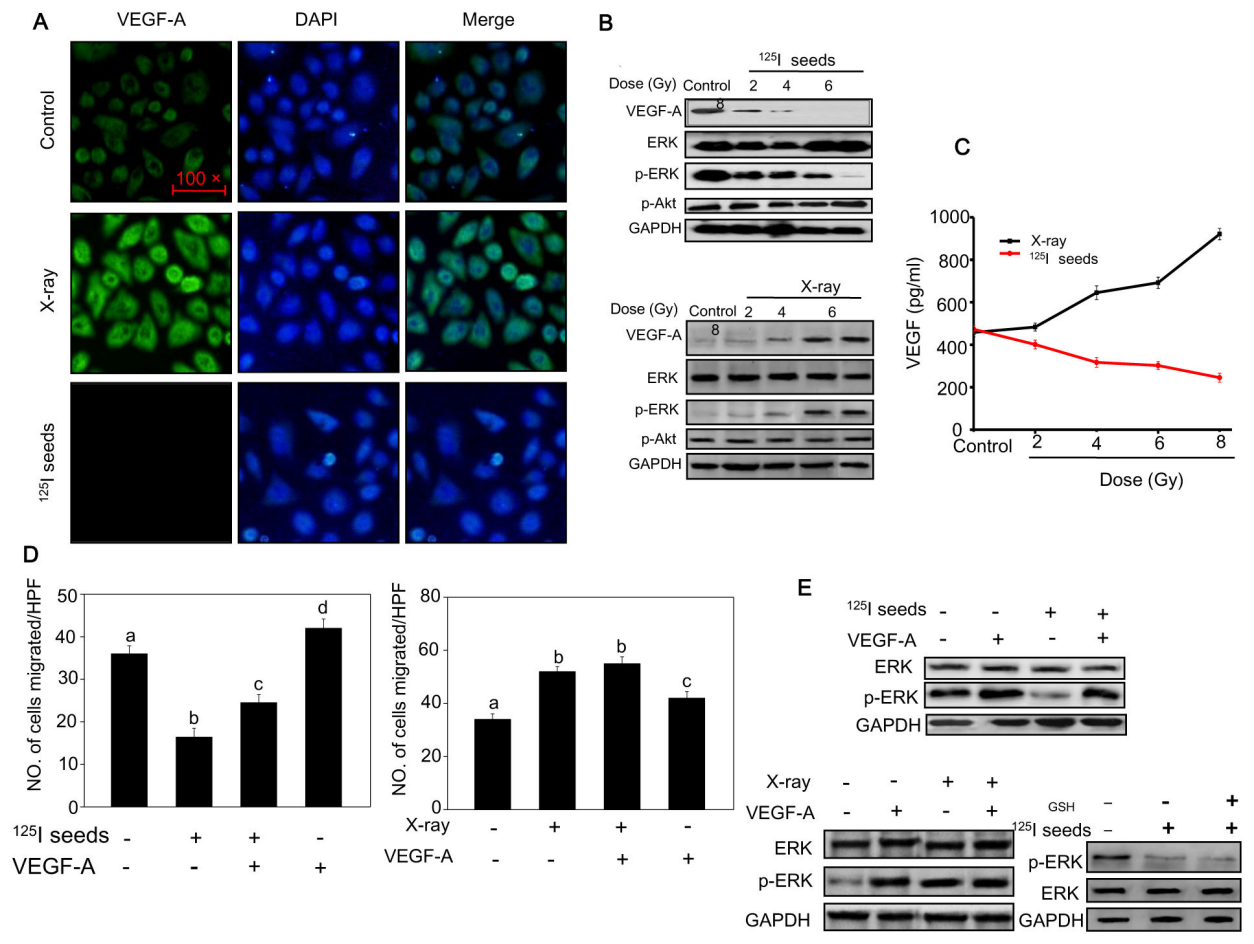
Inactivation VEGF-A/ERK signaling pathway by radioactive ^125^I seeds. (A) Suppression of VEGF-A expression by ^125^I seed irradiation as measured by immunofluorescent assay. (B) Western blotting analysis of the expression levels of VEGF-A/ERK in cells exposed to ^125^I seeds. (C) The extracellular levels of VEGF-A was measured by ELISA. (D, E) VEGF-A (20 ng/ml) significantly blocks the ^125^I seed irradiation-induced inhibition on cell migration by recovering p-ERK protein levels.

VEGF-A plays an important role in cell motility and proliferation. Emerging evidence has confirmed that VEGF-A levels contributed additional prognostic information in head and neck malignancies [16]. Moreover, cell motility is enhanced by the secretion of radiation-induced VEGF-A [18]. Because VEGF-A enhances endothelial cell survival and tumor radioresistance, strategies that target VEGF-A and other endothelial cell survival mechanisms may be used to enhance the cytotoxic effects of radiotherapy [18,24]. Therefore, we first measured VEGF-A expression after irradiation by immunofluorescent assay. As expected, VEGF-A protein levels in cell membrane and cytoplasm decreased significantly in the ^125^I seed irradiation group 24 hours after ^125^I seed irradiation (Figure 6A). Moreover, ^125^I seeds significantly decreased p-ERK levels, but did not affect the Akt pathway (Figure 6B). The effects of irradiation on VEGF-A secretion by NPC cells were also investigated. The results showed that VEGF-A secretion was upregulated by X-ray irradiation. However, VEGF-A secretion was significantly down-regulated by ^125^I seeds irradiation (Figure 6C).

To further confirm the roles of VEGF-A/ERK, we examined the effects of recombinant human VEGF-A on ^125^I seed irradiation-induced inhibition of cell migration. As shown in Figure 6D, we observed a marked increasing number of average migrated cells per high power field (HPF) treated by ^125^I seed from 16.4 to 24.5 after addition of 20 ng/ml human growth factor VEGF-A. We performed western blotting to characterize the role of ERK in cell migration. As shown in Figure 6E, we found that pretreatment of the cells with VEGF-A obviously enhanced ERK activation. Interestingly, the results indicated that pretreatment of cells with GSH could not recover activated ERK levels that were decreased by ^125^I seeds irradiation. Taken together, these results suggest that radioactive ^125^I seeds suppress cell migration with the improvement of VEGF-A/ERK signaling. Moreover, recombinant human VEGF-A could at least partially block the ^125^I seed irradiation-induced inhibition of cell migration by recovering ERK protein levels.

### Radioactive ^125^I seeds exert greater in vivo anticancer activity than X-rays

**Figure 7:**
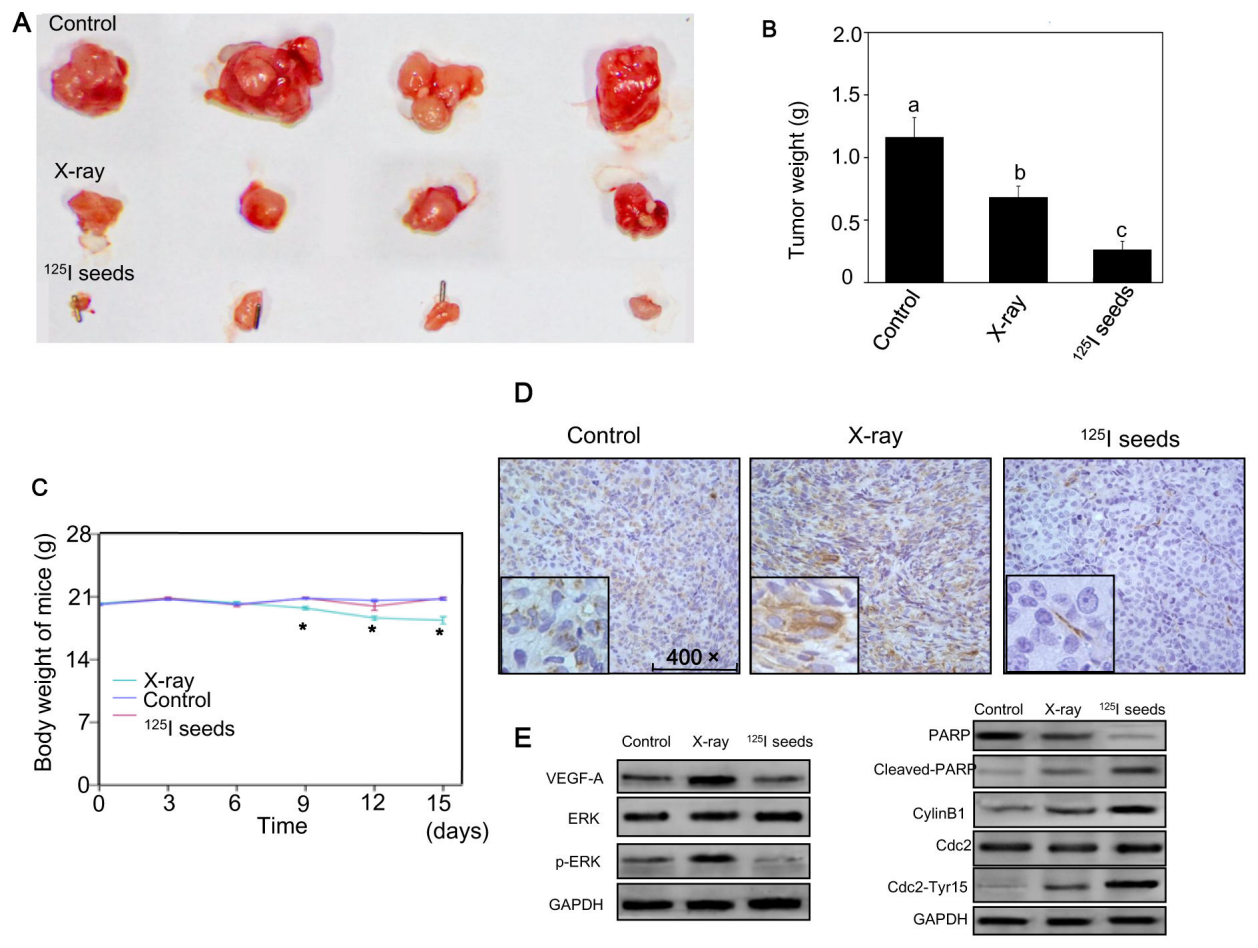
^125^I seed irradiation exhibits greater *in vivo* anticancer **activity than X-ray**. (A) Representative images of tumors treated with ^125^I seed and X-ray irradiation. (B, C) Effects of ^125^I seed and X-ray irradiation on tumor and body weights. Data are presented as mean ± SD (n = 4). Significant difference between ^125^I seed and X-ray groups under the same dose is indicated by * *P*<0.05 and ** *P*<0.01. (D, E) Expression of VEGF-A, p-ERK, Cleaved-PARP and Cdc2/cyclinB1 in xenograft tumors detected by IHC or western blotting.

*In vivo* experiments were also performed to evaluate the effect of ^125^I seed irradiation. In order to maintain consistency with clinical therapy, an animal study for ^125^I seed irradiation was performed according to TPS. The results indicated that X-ray and ^125^I seed irradiation at a cumulative dose of 20 Gy both effectively control the tumor growth. However, the average tumor weight in the ^125^I seeds group was smaller than that of the X-ray group (Figure 7A, B). The average body weight of the nude mice exposed to X-ray irradiation decreased more significantly than that of the ^125^I seed irradiation group (Figure 7C). Moreover, VEGF-A and p-ERK expression in xenograft tumors were increased in the X-ray irradiation group, and decreased in ^125^I seed group as assessed by IHC and western blotting (Figure 7D, E). The same with *in vitro* experiments, cleaved-PARP was increased in both groups. In addition, local hemorrhagic cystitis that was often observed in NPC patients was also found in X-ray irradiated mice, but not in the ^125^I seed irradiation group (data not show), suggesting fewer possible side effects associated with ^125^I seed irradiation.

## Discussion

NPC is one of the most common malignant tumors in Southeast China [25]. Despite the significant clinical advances made in NPC treatment, such as intensity-modulated radiotherapy, the overall survival rate remains unchanged, and many patients die due to local recurrence and resistance to X-ray radiotherapy [3]. Therefore, searching for novel treatments has become a major goal of scientists in the fields of oncology. In this study, we showed that radioactive ^125^I seeds were more effective in inhibiting the growth, migration, and invasion of NPC cells *in vitro* and *in vivo*. Mechanistic studies indicated that the anticancer effect of ^125^I seed irradiation was achieved by triggering DNA damage and inactivating VEGF-A/ERK signaling. This finding provides evidence for the efficacy of ^125^I seeds for treating NPC patients, especially those who experience local recurrence.

We employed colony formation, MTT, EdU and apoptosis assays to evaluate the NPC cell radiosensitivity. The results showed that NPC cells were more sensitive to ^125^I seed irradiation than X-ray irradiation. Similarly, other studies reported that ^125^I seed irradiation induced a significantly higher percentage of apoptosis and lower SF in prostate cancer cells [8,10] and colonic cells by comparing with^60^ CO-γ ray irradiation [9]. To further clarify the mode of cell death induced by ^125^I seed irradiation, treated cells were examined by flow cytometry. The representative DNA distribution histograms demonstrated that ^125^I seeds induced a significantly greater increase in the proportion of cells in apoptosis and G2/M arrest compared to X-ray irradiation. In addition, the western blotting results indicated that ^125^I seeds remarkably enhanced PARP and caspase-3 cleavage. Consistent with our studies, previous reports have described that ^125^I seeds could lead to higher proportions of apoptosis and G2/M arrest in a prostate cell line than X-ray irradiation [9,10].

It is well known that ionizing radiation (IR) primarily leads to single or double-strand DNA breaks (DSBs) that activate DNA damage checkpoints to initiate signals that ultimately lead to a binary decision between cell death and survival. Importantly, irradiation-induced DNA damage directly or indirectly (via ROS) activates the ATM and ATR kinases and then blocks cell cycle progression [19,26,27]. The blocked cell cycle is involved in the complex response of cells to IR to enforce the cell’s fate to live by inducing growth arrest coupled to DNA damage repair or to die by inducing irreversible growth arrest or apoptosis [7] [28]. In this complicated process, the checkpoints are important to ensure the proper sequence of cell cycle events that allow the cells to respond to DNA damage [22]. Therefore, we examined the expression levels of ATM/ATR-Chk1-Cdc2/cyclin B1 because Cdc2/cyclin B1 ultimately regulate the G2/M progression. As expected, we found that ^125^I seeds induced DNA damage and the phosphorylation of ATM, ATR, Chk1 and Cdc2/cyclin B1 in NPC cells in a dose-dependent manner. The level of ROS increased with irradiation dosage. Previous studies have indicated that G2/M arrest induced by DNA damage could slow down cell cycle progression and prevent mitosis. Moreover, it was confirmed that the G2/M checkpoint was an important regulator of radioresistance. Consistent with our findings, CNE2 cells were more sensitive to ^125^I seed irradiation than X-rays, with a higher percentage of G2/M cells detected. This indirectly indicated that ^125^I seeds led to more damaged DNA. According to previous studies, blocking the cell cycle in the G2/M phase without G1/S arrest may be explained as follows: firstly, though the p53-dependent G1/S checkpoint is very sensitive to low damage levels [29], G1/S checkpoint-deficient cells are dependent on G2/M checkpoints for DNA repair [30]. Moreover, the CNE2 cells are an NP tumor cell lines and express a mutant p53 protein [31]. Secondly, a previous study indicated that after full checkpoint activation, the G1/S checkpoint was not necessarily permanent; rather, cells could be released from it, and the G1/S checkpoint was slowly activated. This allowed cells to enter the S phase in the presence of unrepaired DSBs. According to previous studies, cell cycles were tested by flow cytometry 24 hours after irradiation in our experiment, thus, the G1/S checkpoint might not be activated. Studies have indicated that G2/M arrest induced by damaged DNA slows down cell cycle progression and prevents cells from undergoing mitosis. Taken together, these results support the hypothesis that ^125^I seeds trigger DNA damage to induce NPC cell apoptosis and G2/M arrest.

Cell migration and invasion are fundamental components for tumor cell metastasis. Previous studies have reported that a sublethal dose of X-ray irradiation promoted glioma cell migration and invasion in the border area of postoperative radiotherapy [32]. In contrast, other studies reported that both proton and carbon ion irradiation significantly decreased cell migration and invasion [33,34]. Therefore, we employed wound healing, transwell and Boyden assays to further compare the effects of ^125^I seed and X-ray irradiation on NPC cells. Interestingly, we found that X-ray irradiation promoted the NPC cell migration and invasion, while inhibitory effects were observed in the ^125^I seed irradiation group. The VEGF family proteins contain at least seven members, and VEGF-A is a major characteristic of malignant tumors. Overexpression of VEGF-A induces malignant cell motility and proliferation [35]. Therefore, we measured VEGF-A expression in NPC cells exposed to both types of irradiation. The results indicated that the level of VEGF-A decreased in the ^125^I seed irradiation group. Moreover, according to the KEGG signaling pathway (http://www.genome.jp/kegg/) and previous reports, VEGF-A can regulate cell proliferation and migration through ERK activation [36]. As expected, our study confirmed that ^125^I induced a VEGF-A decrease followed by down-regulated phosphorylation of ERK 1/2 with increased irradiation doses. Moreover, after pretreatment with VEGF-A, the migration ability of NPC cells could be rescued and ERK protein expression could be recovered. Moreover, the VEGF family includes other ligands which can also lead to significant induction of cell motility and invasiveness of cancer cells. Based on this and previous evidence, we could conclude that ^125^I seed irradiation inhibits CNE2 cell line migration and invasion by inhibiting VEGF-A/ERK signaling. In contrast, previous studies and our results show that X-ray irradiation can induce ROS overproduction, which up-regulates HIF-1 and finally resulted in increased VEGF-A [37]. Therefore, our results suggest that radioactive ^125^I seeds suppress cell migration by attenuating VEGF-A/ERK signaling pathway.

To date, there are few reports about ^125^I seed irradiation *in vivo*. Therefore, we investigated the anticancer action of ^125^I seed and X-ray irradiation *in vivo*. CT-scanning followed TPS was performed for every animal that underwent ^125^I seed implantation. To determine an accumulative irradiation dose of 20 Gy, about 8 seeds were implanted into mice with approximately 200 mm^3^ tumor volume for 15 days. According to TPS, isodose lines of ^125^I seed irradiation are more conformal to gross tumor volume (GTV), compared with three-dimensional conformal radiotherapy. Interestingly, adjacent tissues were better protected as reflected by dose-volume histogram (DVH) of the abdomen during the experiments. After irradiation for 15 days, X-ray irradiation and ^125^I seed irradiation at a cumulative dose of 20 Gy both effectively inhibited the tumor growth. However, the mean tumor weight in the ^125^I seed group was smaller than that in the X-ray group. Moreover, VEGF-A expression in xenograft tumors was decreased in the ^125^I seed group. The body weight of nude mice exposed to X-ray irradiation was significantly decreased compared to the ^125^I irradiation group. In addition, local hemorrhagic cystitis often observed in NPC patients was also found in X-ray irradiated mice but not in the ^125^I seed irradiation group, suggesting fewer side effects of ^125^I seed irradiation. The *in vivo* experiments results indicate that ^125^I seed irradiation is more effective in eliminating solid tumor and also associated with fewer adverse effects; however, further studies are needed to clarify the underlying molecular mechanisms.

In general, X-rays and gamma rays demonstrate similar biological effectiveness. However, our study and others have confirmed that ^125^I seeds treatment has greater tumor killing effect compared with conventional X-ray irradiation under the same physical dose [9,10,38]. In our opinion, this may be due to several reasons. Firstly, it can be speculated that if the dose-rate is low, then repair mechanisms are not optimally triggered and the cells remain in a sensitive state. Secondly, the absorption of ionizing radiation by living cells can directly disrupt atomic structures or act indirectly through water radiolysis, thereby generating ROS. As shown in our results, ^125^I seeds induced higher levels of ROS than X-ray irradiation which might lead to more DNA damage. Furthermore, the long accumulation time for a certain dose when given at low dose rate has been assumed to be the cause of the tumor killing effect exhibited by continuous ^125^I seeds irradiation. When the duration of the irradiation is long or continuous (e.g. ^125^I seeds), there is no time for repair or possibly repopulation during irradiation. However, there is a time for repair during between fractions for fractional irradiation (e.g. X-ray). Consistent with our study, the same effects are achieved in ^125^I seed and X-ray groups at a dose of 2 Gy, but ^125^I seeds are more effective after 4 Gy irradiation. Finally, we confirmed that the invasion which enhanced by X-ray irradiation could be inhibited by ^125^I seed irradiation via decreased VEGF-A/ERK signaling.

**Figure 8:**
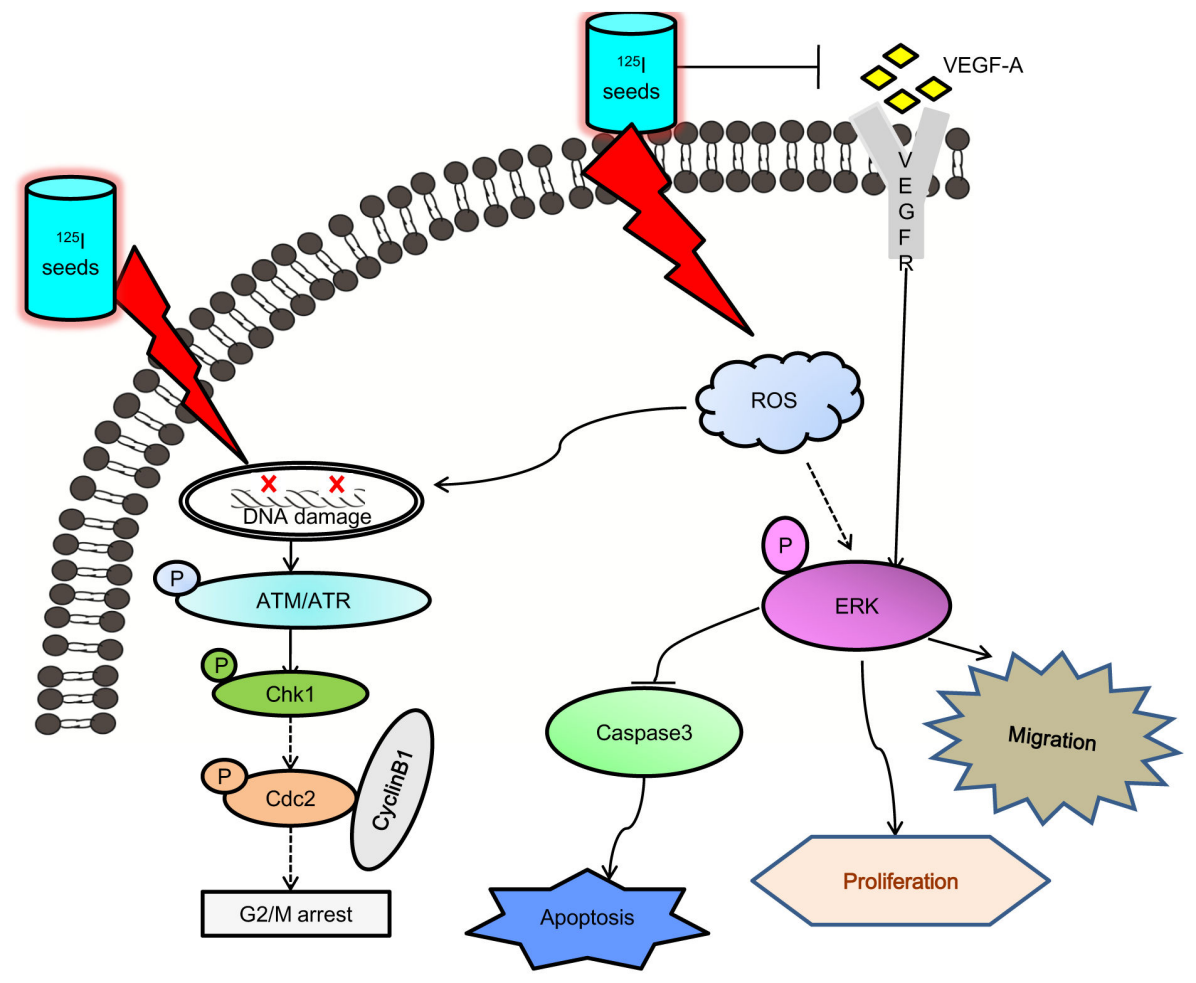
Proposed signal pathways of apoptosis and cell cycle arrest induced by ^125^I seeds. ^125^I seeds caused DNA damage to activate the sensory ATM/ATR kinases, finally results in cell apoptosis and G2/M arrest. At the same time, ^125^I seeds inhibit cells migration by inactivation VEGF-A/ERK pathway. VEGF-A which can increase p-ERK levels was inhibited by ^125^I seeds to regulate cellular proliferation, survival and migration.

In summary, we have demonstrated for the first time that radioactive ^125^I seeds are more effective than X-ray irradiation in inhibiting NPC cell growth through inducing apoptosis triggered by DNA damage. Moreover, cell migration was effectively inhibited by ^125^I seed irradiation, which inactivated VEGF-A/ERK. Pretreatment of cells with VEGF-A significantly blocked the ^125^I seed irradiation-induced inhibition of cell migration by recovering ERK protein levels. Notably, the *in vivo* findings confirmed that ^125^I seed irradiation was more effective in inhibiting tumor growth than X-ray irradiation. Taken together, these results suggest that radioactive ^125^I seeds exhibit novel anticancer activity by triggering DNA damage and inactivating VEGF-A/ERK signaling (Figure 8). This finding provides evidence for the efficacy of ^125^I seeds for treating NPC patients, especially those who experience local recurrence.
